# The elevation of miR-185-5p alleviates high-fat diet-induced atherosclerosis and lipid accumulation *in vivo* and *in vitro* via SREBP2 activation

**DOI:** 10.18632/aging.203896

**Published:** 2022-02-16

**Authors:** Wenyun Tan, Gang Wang, Gang Liu, Daofeng You, Mei Wei, Xiaojing Jin, Wei Zhao, Mingqi Zheng

**Affiliations:** 1 Department of Cardiology, The First Hospital of Hebei Medical University, Shijiazhuang, Hebei, China; 2 Department of Cardiology, 980 Hospital of PLA Joint Logistics Support Forces, Shijiazhuang, Hebei, China; 3 Department of Emergency, The First Hospital of Hebei Medical University, Shijiazhuang, Hebei, China; 4 Department of Ultrasound, The First Hospital of Hebei Medical University, Shijiazhuang, Hebei, China

**Keywords:** miR-185-5p, ox-LDL, SREBP2, atherosclerosis, lipid metabolism

## Abstract

Objective: SREBP2, a member of the *SREBP* family, is a primary regulator of lipid metabolism. In recent years, an increasing number of studies have suggested that miRNAs regulate lipid metabolism-related genes. It was speculated in this study that miRNAs may be implicated in the regulation of lipid accumulation in macrophages by SREBP2 protein.

Methods and results: GSE34812, GSE132651 and GSE28829 datasets comprised of atherosclerosis samples were downloaded to explore the gene expression profiles related to the miRNAs and SREBP2, and miR-185-5p was predicted to be a target of SREBP2. The GO annotations and KEGG pathway analysis were adopted for functional classification of differentially expressed genes, and lipid metabolic process was an enriched pathway in atherosclerosis. Besides, the effects of SREBP2 on increasing lipid accumulation were investigated *in vivo* using miR-185-5p mimic/apoE^−/−^ mice and miR-185-5p NC/apoE^−/−^ mice. All mice fed with a HFD suffered from atherosclerosis. Moreover, the effects of miR-185-5p on atherosclerotic plaque formation in mice were analyzed. An *in vitro* assay was also performed to determine the effect of miR-185-5p on ox-LDL-stimulated RAW 264.7 macrophages. Finally, miR-185-5p mimic was transfected into cultured macrophages. The results showed that the miR-185-5p elevation might regulate lipid accumulation in mice by targeting SREBP2. Furthermore, miR-185-5p mimic repressed the activation of SREBP1, SREBP2, LDLR, SCD-1, HMGCR as well as NLRP3, IL-1β, TNF-α in HFD fed mice or ox-LDL-stimulated macrophages.

Conclusions: our study demonstrated that miR-185-5p effectively alleviates atherosclerosis and lipid accumulation by regulating the miR-185-5p/SREBP2 axis.

## INTRODUCTION

Atherosclerosis is an inflammatory disease caused by a hypercaloric high-fat diet (HFD) and primarily intermediated by macrophage infiltration into the arterial wall [1, 2]. It is the leading cause of morbidity and mortality worldwide, characterized by the formation of arterial plaques [3, 4]. Besides, atherosclerosis leads to most cardiovascular disorders, including acute myocardial infarction, and contributes to the increase of cardiovascular deaths [5, 6]. It has been reported that increased lipid accumulation is a crucial player in atherosclerosis progression [7, 8]. Oxidized low-density lipoprotein (ox-LDL) is validated to be able to induce inflammatory responses during atherosclerosis formation and progression [9]. Despite significant advancement in therapeutic methods for atherosclerosis, the disease remains the primary cause of morbidity and mortality around the world. Therefore, it is urgently needed to explore the molecular mechanisms in atherosclerosis and identify novel targets for treatments.

Micro RNAs (miRNAs) are a class of small, endogenous, non-coding RNAs with 21-24 nucleotides in length, which can bind to the 3′ untranslated region (3'-UTR) of their target mRNAs and participate in the regulation of many biological processes, such as cell differentiation, proliferation, homeostasis, angiogenesis, and tumorigenesis [10–12]. Furthermore, miRNAs have been proven as significant players in atherosclerosis development. For example, Schober et al. indicated that miR-126-5p prevented atherosclerotic lesion formation by regulating EC turnover [13]. Meanwhile, miR-15a is found to be an anti-inflammatory factor for atherosclerosis-associated foam cell formation [14]. Yébenes et al. manifested that inhibiting miR-217 in the vasculature could partially protect vascular function and relieve atherosclerosis [15]. In addition, Zhang et al*.* discovered that the therapy targeting miR-155 combined with existing conventional therapies might serve as an effective treatment for atherosclerosis [16]. Zhang et al. revealed that treatment with antisense oligonucleotides against miR-144 might enhance reverse cholesterol transport and oxysterol metabolism in patients with cardiovascular disease [17]. Furthermore, Wang et al. uncovered that miR-296 played a significant role in the regulation of atherosclerosis [18]. Earlier studies have indicated that miR-185-5p acts as a tumor suppresser, and it is expressed in many human cancers [19–21]. However, the molecular mechanism of miR-185-5p in atherosclerosis remains uncertain. Sterol response element-binding protein 2 (SREBP2), a member of the *SREBP* family, is a crucial regulator of lipid metabolism [22, 23]. In recent years, an increasing number of studies have suggested that miRNAs are involved in regulating lipid metabolism-related genes [24]. In the present study, the assumed role of miR-185-5p in regulating the lipid metabolism and its underlying mechanisms in atherosclerosis were studied, so as to provide novel strategies for atherosclerosis treatment.

## MATERIALS AND METHODS

### Microarray data analysis

The gene expression profiles in GSE34812, GSE132651, and GSE28829 datasets were downloaded from Gene Expression Omnibus (GEO) database. The differentially expressed genes (DEGs) were acquired through differential analysis with |logFC|>2 and padj<0.05. Next, overlapping DEGs obtained from the microarray analysis were depicted into Venn diagrams using the online Venn Diagram tool, and the key genes were recognized. The assumed miRNAs targeting the DEGs were predicted by TargetScan, miRDB, miRWalk, and DIANA tools. The DEGs among the GSE34812, GSE132651, and GSE28829 datasets were identified by the intersect function in the R package.

### Human specimens

All human specimens of carotid atherosclerotic plaques were collected form patients of the first affiliated hospital of Hebei Medical University, and all the experimental operations were under the ethics, moreover, the detailed information about these patients were shown in Supplementary Table 1.

### Mice

Eighteen male apoE^−/−^ mice on the C57BL/6J background, aged 6-8 weeks old, were purchased from the Experimental Animal Center of Skbex Biotechnology. The mice were fed with HFD (18% fat and 1.25% cholesterol) for 3 months as previously described to induce atherosclerosis [13]. All animal experiments were approved by the Ethics Committee of the First Hospital of Hebei Medical University. Subsequently, the mice were randomly classified into two groups, namely miR-185-5p mimic group and miR-185-5p negative control (NC) group, with 9 mice in each group, and then they were intravenously injected with 100 nM miR-185-5p mimic and NC miRNAs, respectively, via the caudal veins every other day according to Ye Yao's methods [25]. The intervention results were detected 12 weeks later. Finally, the mice were euthanized by intraperitoneally injected pentobarbital sodium (50 mg/kg).

### Oil Red O staining and pathological evaluation and hepatic, serum cholesterol levels

Abdominal vascular tissues were harvested from the mice, which were longitudinally dissected, frozen immediately and rinsed in PBS for three times, followed by soaking in 4% paraformaldehyde for 20 min and Oil Red O staining (Service Biology, Wuhan, Hubei, China) at room temperature for 2 h. Next, images were visualized using a Nikon Eclipse 80I microscope equipped with a Nikon DS-EI1 camera (Nikon, Japan). The frozen aortic root sections were stained with Oil Red O to evaluate the atherosclerotic plaque areas, and immunohistochemical staining for MAC-3 and α-SMA was used to analyze the areas of lesional macrophages and smooth muscle cells (SMCs). Moreover, the Sirius Red staining and polarized light photography were employed to assess the collagen content. The hepatic homogenate as well as serum cholesterol levels were tested by the mice total cholesterol (TC) ELISA detection kit (EK-Bioscience, Shanghai, China).

### Western blotting analysis

Tissues and cells were prepared for Western blotting analysis as described in previous study [26]. Primary and secondary antibodies were applied to incubate the tissues and cells at room temperature for 2 h. Specifically, β-actin (1:5000 dilution) was taken as the control of proteins derived from tissues and cells. All the antibodies against SREBP1 and SREBP2 (1:1000 dilution), LDL receptor (LDLR) (1:1000 dilution), Fas (1:2000 dilution), stearoyl-CoA desaturase 1 (SCD-1), and 3-Hydroxy-3-Methylglutaryl-CoA Reductase (HMGCR) were purchased from Abcam. Finally, the proteins were detected using an enhanced chemiluminescence (ECL) western blotting kit (Amersham Life Sciences, UK).

### Preparation of nuclear extracts from atherosclerotic tissues

The tissue suspension was centrifuged at 3000 g for 5 min. The precipitate was resuspended with 5 mL of homogenate, transferred into a mortar and ground into homogenate. Then the homogenate was filtered twice with double-layer Miracloth, transferred into a 15 mL Falcon tube and centrifuged at 1000 g for 10 min. After that, the precipitate was gently resuspended with homogenate and placed on the top of 2.0 mol/L sucrose gradient solution containing 37.5 mmol/L Tris-maleic acid (pH 6.5), 5 mmol/L MgCl2 and 1% Dextrin T500, followed by centrifugation at 50000 g and 4° C for 30 min. After the supernatant was carefully removed, the precipitate was gently resuspended with 5 mL of homogenate again, and the resuspension was placed on the top of the 2.0 mol/L sucrose gradient solution and centrifuged at 50000 g for 30 min. Finally, the precipitates containing nuclei were resuspended using 100 nM homogenate, and the suspension containing nuclei was used to extract nuclear proteins.

### Cell culture and treatment

RAW 264.7 cells were maintained in DMEM (Thermo Fisher Scientific, USA) containing 10% fetal bovine serum (FBS, Wisent, Canada), 100 μg/mL penicillin, and 100 g/mL streptomycin sulfate. After reaching 80-90% confluence, RAW 264.7 cells were transfected in accordance with the instructions of Lipofectamine 2000 (11668-019, Invitrogen, Carlsbad, CA, USA). Subsequently, RAW 264.7 cells were co-transfected with 75 μg/mL ox-LDL (YB-002, Yiyuan Biotech, Guangzhou, Guangdong, China), miR-185-5p NC, miR-185-5p mimic or miR-185-5p inhibitor. The SREBP2-overexpression (OE) lentivirus, miR-185-5p mimic, and miR-185-5p inhibitor were obtained from Beijing Syngentech Co., Ltd. (Beijing, China).

### Immunofluorescence staining

Cell climbing sheets were gathered, washed with PBS for three times, fixed in 4% paraformaldehyde for 30 min at room temperature, and permeabilized with 0.1% Triton X-100 and blocked with 5% BSA plus Tween 20. Then, the sections were incubated with anti-SREBP2 antibody (diluted at 1:100, Abcam) overnight at 4° C, followed by 1 h of incubation with secondary antibodies at room temperature. Next, the nuclei were counterstained with DAPI (diluted at 1:1,000, Thermo Fisher Scientific) for 10 minutes at room temperature. Finally, fluorescence intensity on the stained sections of aortic roots was measured using ImageJ software.

### Detection of phagocytosis of latex beads by RAW 264.7 cells

Mouse macrophage cell line RAW 264.7 infected with NC, mimic, or inhibitor of miR-185-5p was plated on a glass coverslip and incubated with 75 μg/mL ox-LDL as well as 1-μm Fluoresbrite YG Microsphere latex beads or microspheres (Polysciences; 4.55 × 10^6^ beads per mL) for 24 hours. Then the coverslip with cells were stained with DAPI for visualization of nuclei and Dil for visualization of membrane according to the instructions. Digital images were captured under a fluorescent microscope at different excitation wavelengths to identify the latex beads for further quantitative analysis of the numbers of latex beads phagocytized by macrophages. The number of latex beads and nuclei were counted in five randomly selected fields under each experimental condition. The phagocytotic activity was expressed as the number of latex beads divided by the number of nuclei in the fields [27].

## RESULTS

### Implications of SREBP2 and miR-185-5p in atherosclerosis

**Figure 1:**
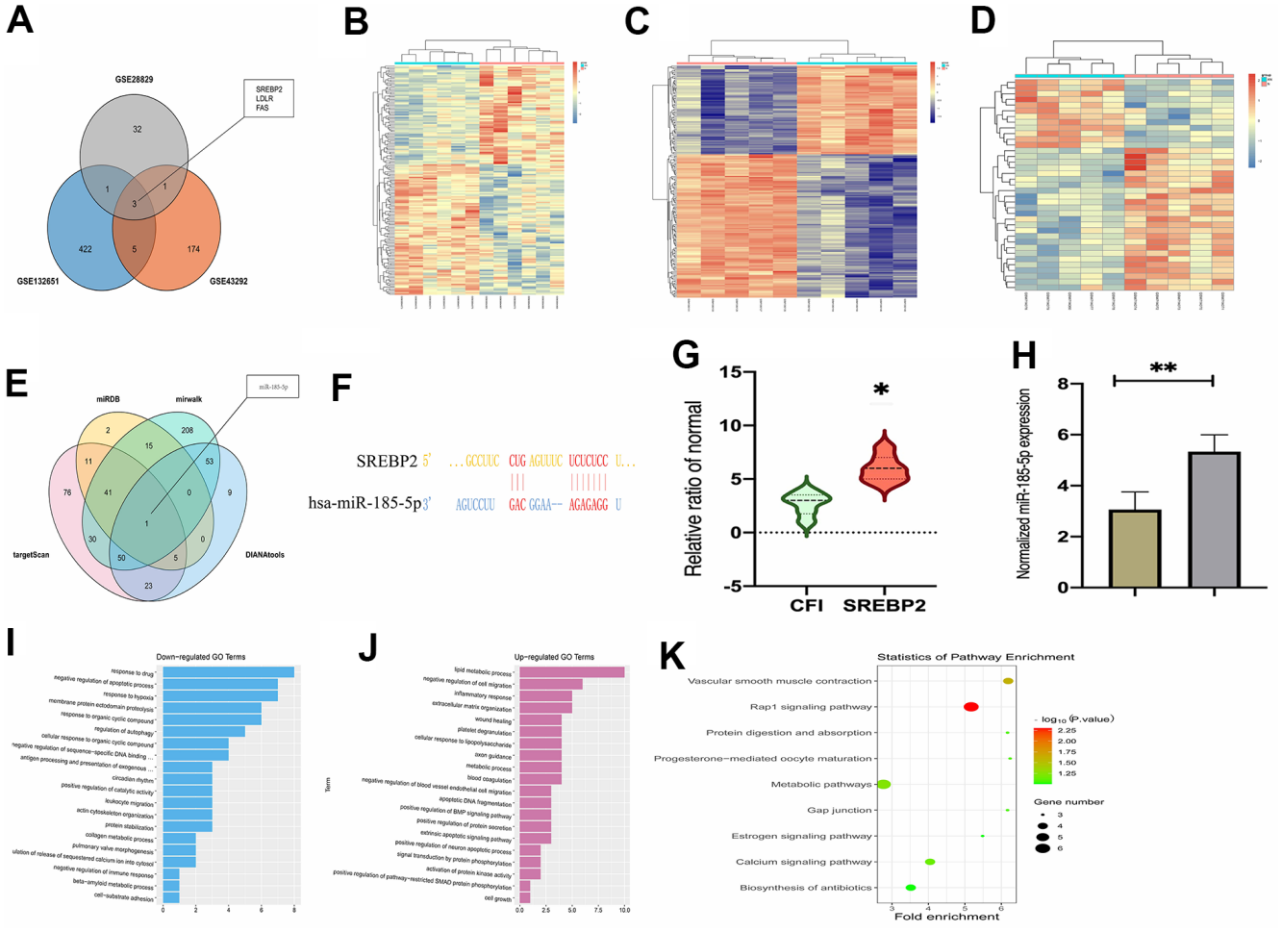
**MiR-185-5p was predicted as a candidate miRNA that affects atherosclerosis progression through regulating SREBP2.** (**A**) Venn diagram of the overlap of the top 100 DGEs among GSE43292, GSE132651, and GSE28829 datasets; (**B**) a heat map depicting the top 100 DEGs from the GSE132651 dataset; (**C**) the expression of SREBP2 in atherosclerosis in the GSE132651 dataset (P<0.05); (**D**) the top 50 miRNAs predicted from four web-based resources and miR-185-5p exhibited the highest statistical score (P<0.05); (**E**) the sequence of miR-185-5p binding to SREBP2 predicted using the biological prediction online resource; (**F**, **G**) the enrichment processes of GO annotations and lipid metabolic process were enriched pathways in atherosclerosis; (**H**) miR-185-5P levels from human specimens were tested by Q-PCR; (**I**, **J**) GSEA enrichment analysis shows that it is enriched in lipid metabolic signaling pathway, (**K**) Results of KEGG pathway analysis showed all differential genes.

To identify the mRNAs that may contribute to the atherosclerosis progression, the gene expression profiles in GSE43292, GSE132651, and GSE28829 datasets were obtained from the GEO database comprised of atherosclerosis samples, and samples with insufficient clinical data were ignored. The top 100 DEGs in these datasets were obtained. Then Venn diagram analysis was carried out to visualize the overlapping DEGs among these datasets, including SREBP2, LDLR, and Fas (Figure 1A). A heat map depicting the top 50 DEGs from GSE132651 dataset was shown in Figure 1B. A heat map depicting the DEGs from GSE43292 dataset was displayed in Figure 1C. A heat map depicting the DEGs from GSE28829 dataset was exhibited in Figure 1D. Next, the assumed miRNAs targeting SREBP2 were determined by TargetScan, miRDB, miRWalk, and DIANA tools. By evaluating the top 50 miRNAs from each prediction website, miR-185-5p was identified as the common predictive miRNA. Besides, SREBP2 and miRNA were predicted respectively, and Wayne diagram was used to obtain the intersection (Figure 1E). In particular, it was found through comparing the prediction results that miR-185-5p had targeted binding regions with SREBP2 (Figure 1F). SREBP2 was identified as a highly expressed gene in atherosclerosis according to the data from GSE132651 dataset (Figure 1G), suggesting that SREBP2 may affect the development of atherosclerosis. The miR-185-5p levels were tested by Q-PCR in atherosclerotic plaques and surrounding tissue, and the miR-185-5p levels were much lower in AS plaques (Figure 1H). To further explain gene expression alterations in atherosclerosis, functional classification of DEGs was performed by means of the Gene Ontology (GO) annotations and Kyoto Encyclopedia of Genes and Genomes (KEGG) pathway analysis. According to the data from GSE132651 dataset, the enrichment processes of GO annotations were depicted in Figure 1I, 1J. It was manifested that the DEGs were mainly enriched in the lipid metabolic process, inflammatory response, and metabolic process. All differential genes discovered via KEGG pathway analysis were depicted in Figure 1K. The above results implied that the effects of miR-185-5p and its target gene SREBP2 on atherosclerosis may be realized via the lipid metabolic signaling pathway.

### MiR-185-5p mimic increased serum but decreased hepatic cholesterol levels and suppressed atherosclerosis in HFD-fed apoE^-/-^ mice

**Figure 2:**
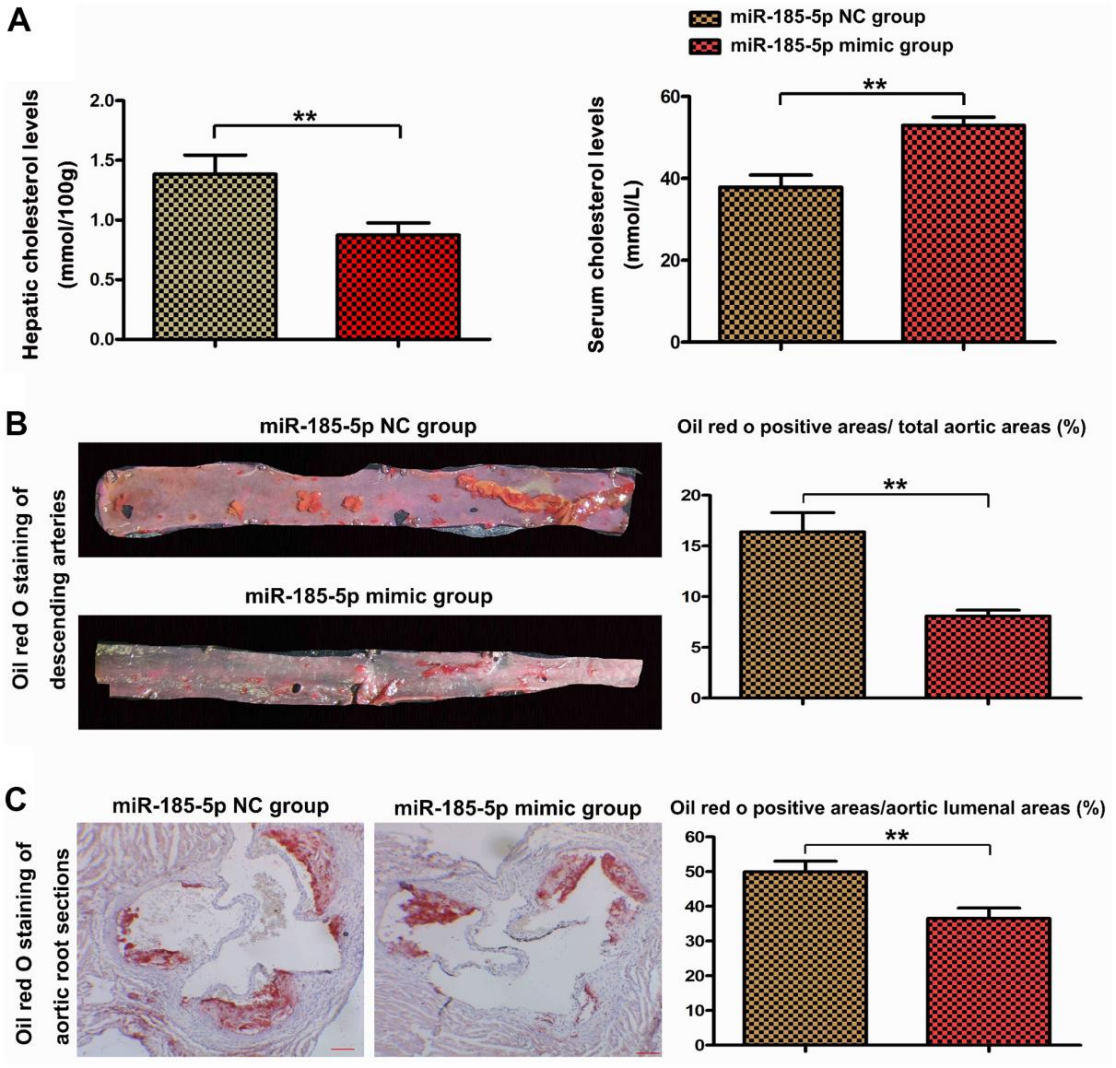
**MiR-185-5p mimic decreased atherosclerotic lesions in HFD-fed apoE^−/−^ mice.** To induce atherosclerosis, apoE^−/−^ mice fed a high-fat diet were administered with miR-185-5p mimic via tail vein once every 2 weeks for 12 weeks. (**A**) hepatic cholesterol levels and serum cholesterol levels, (**B**) The descending arteries were obtained from the apoE^−/−^ mice and stained with Oil-red-O to examine the plaque formation. (**C**) The longitudinal section of the mice's aortic root was stained with Oil red O to examine the lesion area.

Increased serum cholesterol levels and decreased hepatic cholesterols levels were found in miR-185-5p mimic mice compared with miR-185-5p NC group, results were showed in Figure 2A. To investigate the effects of miR-185-5p on atherosclerosis, we fed a high-fat diet to apoE^-/-^ mice to induce atherosclerosis. As shown in Figure 2B, miR-185-5p mimic was found to diminish plaque formation in descending arteries of apoE^-/-^ mice by Oil-red-O staining. The quantitative analysis further confirmed that miR-185-5p mimic mice exhibited lower plaque ratios (positive area/total aortic area) than those in the NC group (p<0.05). To further evaluate the effect of miR-185-5p, we performed Oil-red-O staining to the longitudinal dissection of the aorta from the miR-185-5p mimic and NC group. As shown in Figure 2C, the aorta root-lesion area demonstrated that miR-185-5p mimic significantly decreased the lesion area. Furthermore, miR-185-5p mimic also resulted in decreased plaque ratios (positive area/aortic luminal areas) observed in Oil-red-O-stained aortic root sections when compared to those of the NC group (P<0.05). These results implied that miR-185-5p mimic decreased atherosclerosis in HFD-fed apoE^-/-^ mice.

### MiR-185-5p mimic down-regulates lipid metabolic gene as well as inflammatory factors expression in HFD-fed apoE^-/-^mice

**Figure 3:**
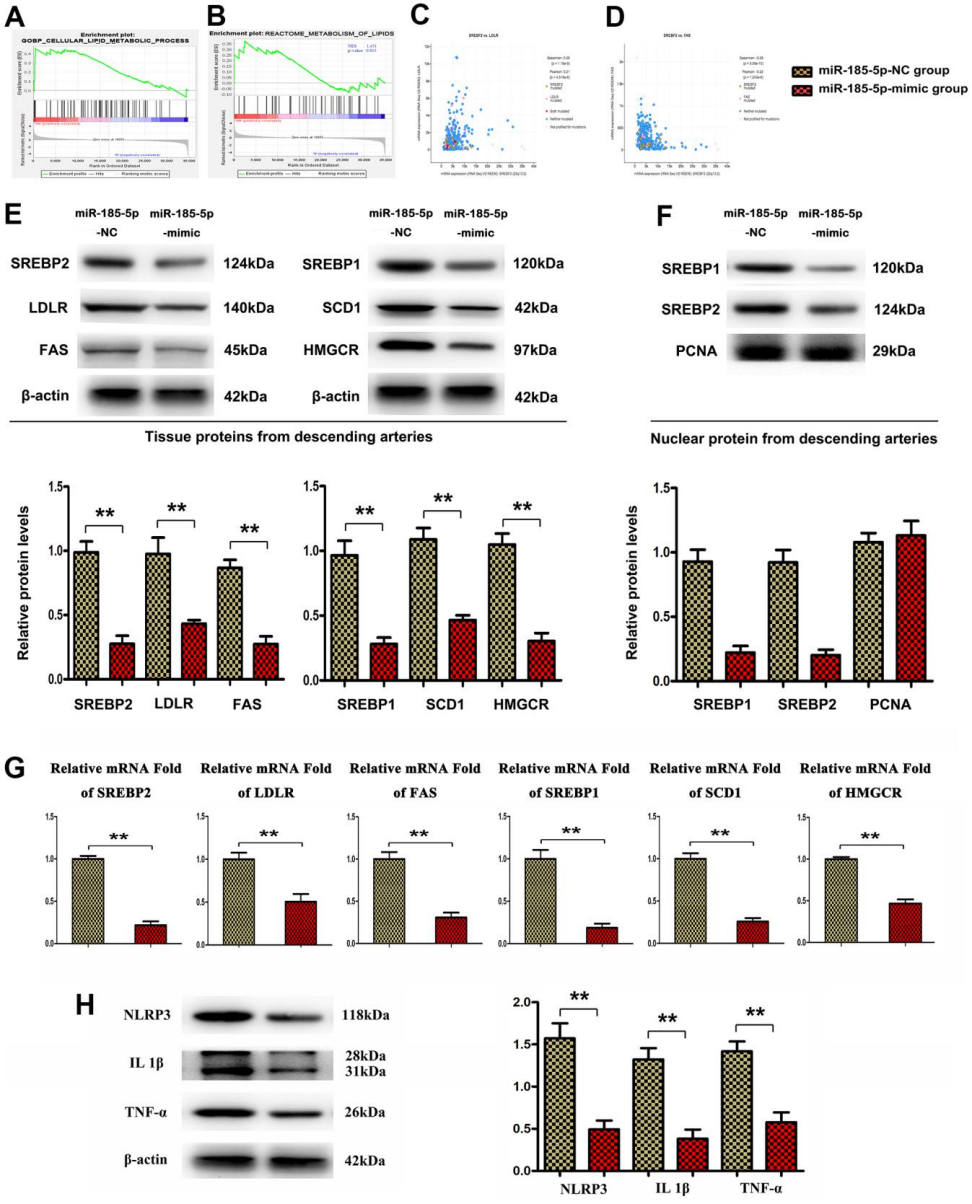
**MiR-185-5p mimic inhibited lipid metabolic gene expression in HFD-fed apoE^-/-^ mice.** (**A**, **B**) GSEA enrichment analysis shows that it is enriched in lipid metabolic signaling pathway. (**C**, **D**) co-expression analysis revealed that LDLR and FAS were significantly correlated with SREBP2 in atherosclerosis. (**E**, **G**) Western blotting and Q-PCR analysis was conducted to assess the protein levels of LDLR, FAS, and SREBP2, SREBP1, SCD1, HMGCR, NLRP3, IL-1β, TNF-α in the aortic sections of HFD-fed apoE^-/-^ mice. (**F**) nuclear protein levels of SREBP1 and 2. (**H**) western blotting analysis to test the protein levels of NLRP3, IL-1β, TNF-α. (*,P <0.05; **, P <0.01).

To examine the effects of MiR-185-5p on lipid metabolism at the molecular levels, the expression of significant regulators involved in lipid metabolic processes was evaluated. Firstly, GSEA enrichment analysis predicted that the abnormal regulation of core differential genes was related to some important biological processes. The annotated gene sets were c2.cp.reactome.v7.4.symbols.gmt and c5.go.bp.v7.4symbols.gmt. According to the P value, the pathways with significant difference enrichment were screened, and the pathways inhibited by two genes were selected as the possible pathways. The results suggest that it is enriched in signaling pathways such as lipid metabolism pathway (Figure 1A, 1B). Furthermore, co-expression analysis revealed that LDLR and FAS were significantly correlated with SREBP2 in atherosclerosis (Figure 3C, 3D). We then examined the mRNA and protein expression of LDLR, FAS, and SREBP2 as well as SREBP1, SCD1, HMGCR, the lipid metabolic proteins [25, 26, 28, 29], in the lesions of atherosclerotic aortas from apoE-/- mice with HFD in tissue as well as nuclear proteins, shown in Figure 3E, 3F. The SREBP2 directly combine with the activation of NLRP3, the powerful inflammation activator in AS, therefore the associated inflammatory factors IL-1β, TNF-α were also tested by western blot. The miR-185-5p mimic inhibited these lipid biosynthesis and inflammatory factors vs miR-185-5p NC mice, data were shown in Figure 3E–3G (P<0.05).

### MiR-185-5p inhibited lipid metabolism pathway by repressing SREBP2 activation

**Figure 4:**
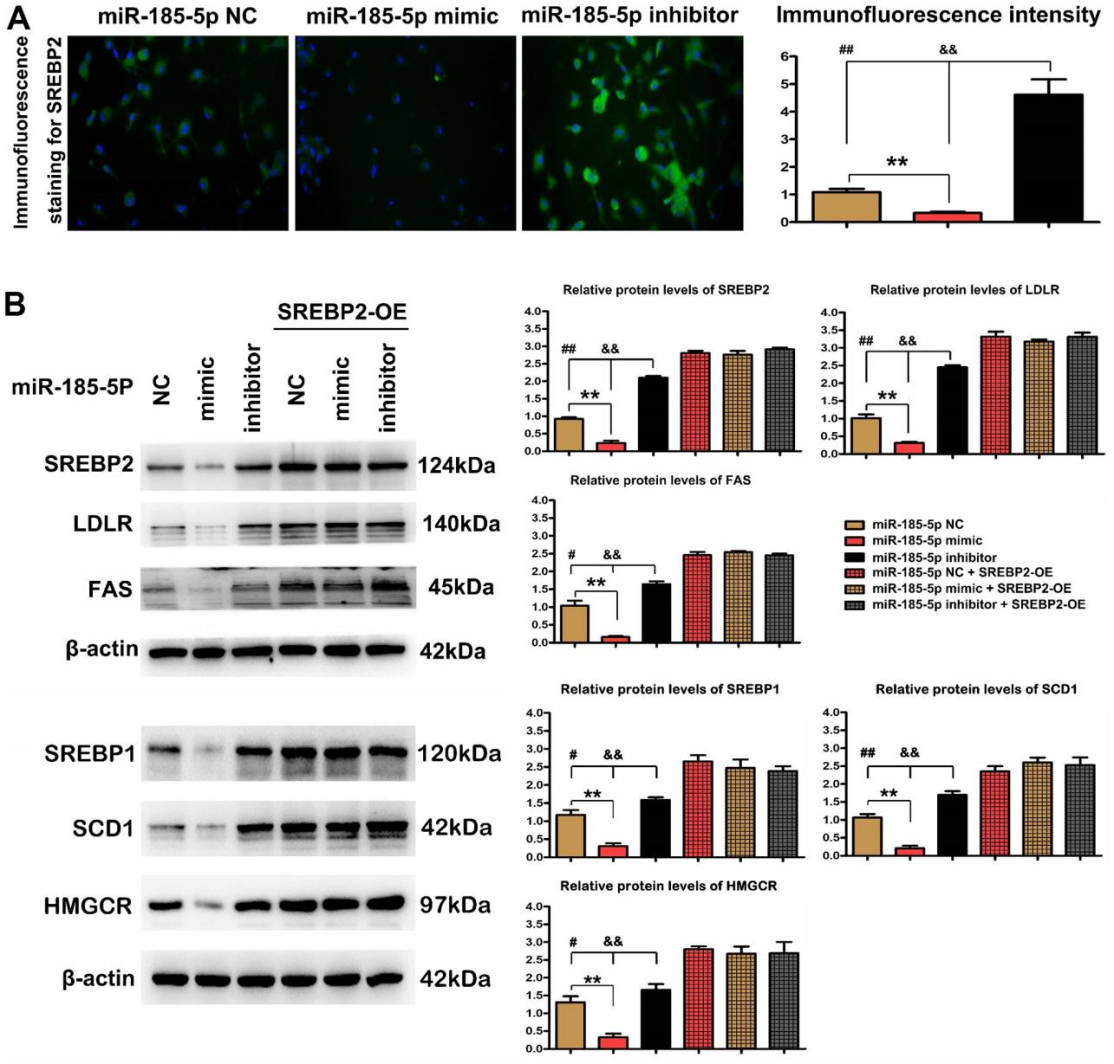
**MiR-185-5p inhibited SREBP2 activation and thus repressed lipid metabolism pathway.** Macrophages were pretreated with ox-LDL stimulation. (**A**) Immunohistochemical staining of SREBP2 in miR-185-5p mimic group, inhibitor group, and negative control group; the positive expression rate of SREBP2. *P<0.05 in miR-185-5p mimic, inhibitor vs. NC groups. (**B**) Western blotting analysis of SREBP2, LDLR, and FAS in miR-185-5p NC group, miR-185-5p mimic group, or miR-185-5p inhibitor group. The SREBP2, LDLR, and FAS, SREBP1, SCD1, HMGCR protein levels were measured after co-transfection with miR-185-5p mimic, miR-185-5p NC, or miR-185-5p inhibitor, and SREBP2-OE Lentivirus in ox-LDL stimulated macrophages. Different data were presented as mean ± S.D. (*,P <0.05; **, P <0.01).

Next, the effect of miR-185-5p on SREBP2 *in vitro* was particularly studied. The macrophage cell line RAW 264.7 was treated with ox-LDL as well as NC, mimic, and inhibitor of miR-185-5p. The positive expression of SREBP2 was increased in macrophages exposed to ox-LDL. It was revealed by immunofluorescence staining that the administration of miR-185-5p mimic significantly down-regulated the expression of SREBP2 in ox-LDL-stimulated macrophages, whereas the decreased expression of SREBP2 was significantly recovered after pretreatment with miR-185-5p inhibitor (Figure 4A). Subsequently, it was demonstrated by quantitative analysis that the immunofluorescence intensity of SREBP2 in ox-LDL-stimulated macrophages was decreased in miR-185-5p mimic group compared with that in miR-185-5p NC group and miR-185-5p inhibitor group (P<0.05). Western blotting analysis confirmed that miR-185-5p mimic reduced the protein expressions of SREBP2, LDLR, and Fas as well as SREBP1, SCD1, HMGCR in ox-LDL-stimulated macrophages. The results were further supported by the quantitative analysis on relative protein levels. Compared with those in miR-185-5p NC group and miR-185-5p inhibitor group, the protein expressions of SREBP2, LDLR and Fas as well as SREBP1, SCD1, HMGCR were significantly down-regulated in miR-185-5p mimic group (P<0.05). These results suggested that miR-185-5p mimic specifically inhibits the activation of lipid metabolism-related genes in ox-LDL -stimulated macrophages. Furthermore, the effects of SREBP2-OE Lentivirus in ox-LDL-stimulated macrophages were examined, and the results indicated that SREBP2-OE Lentivirus significantly restored the decreased protein expression of SREBP2, LDLR, and Fas in miR-185-5p mimic group (Figure 4B), suggesting that miR-185-5p regulates the protein levels of lipid metabolism-related genes through SREBP2 in ox-LDL-stimulated macrophages. In summary, these results suggest that miR-185-5p inhibits the protein levels of lipid metabolism-related genes through SREBP2 in ox-LDL-stimulated macrophages.

### Effect of MiR-185-5p mimic on lipid accumulation in macrophages

**Figure 5:**
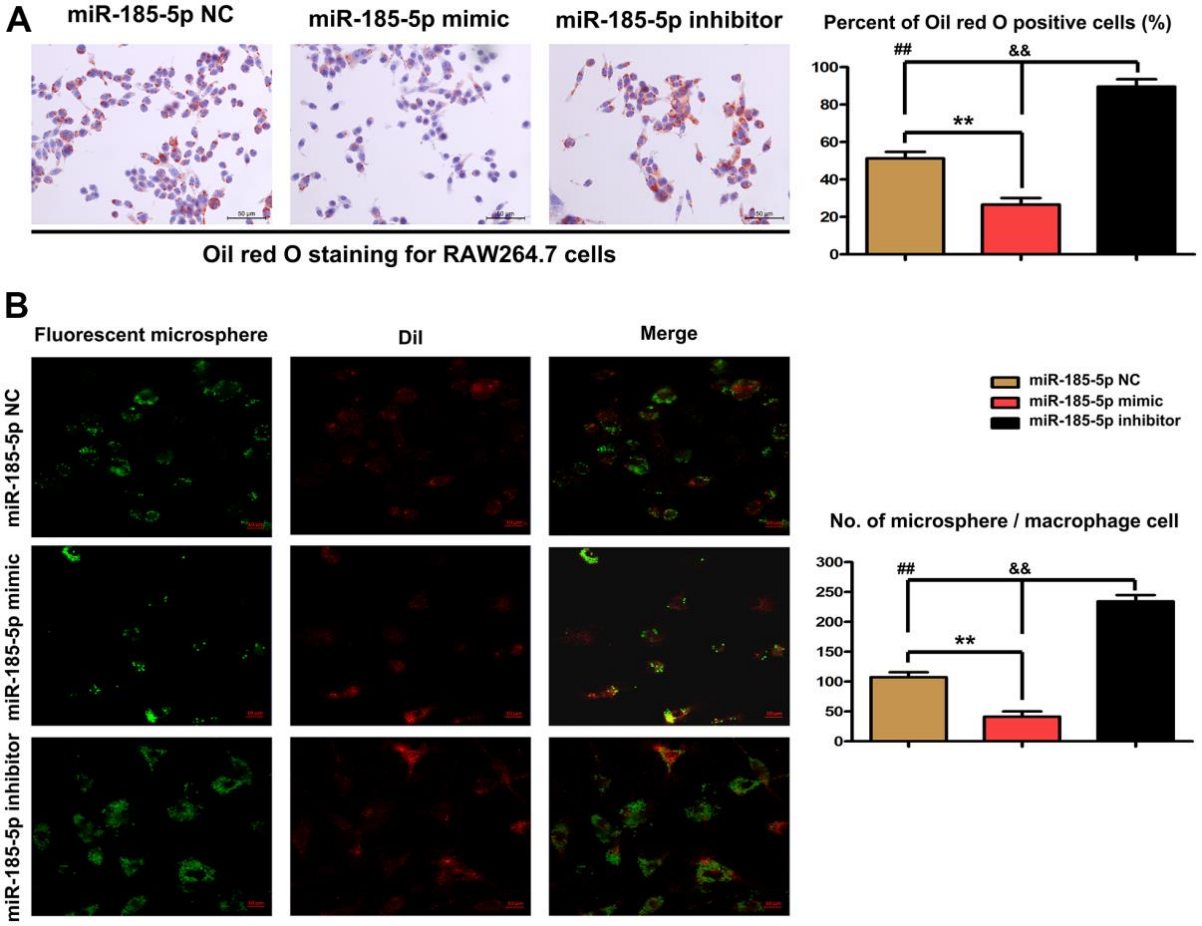
**Effect of miR-185-5p mimic on lipid accumulation in macrophages.** Macrophages were pretreated with ox-LDL stimulation. (**A**) Oil Red O staining revealed that co-treatment with miR-185-5p mimic and ox-LDL significantly decreased the number of lipid droplets. MiR-185-5p inhibitor markedly increased the number of lipid droplets in macrophages. (**B**) The microsphere phagocytosis assays revealed that significantly fewer fluorescent microspheres infiltrated into the macrophages in miR-185-5p mimic group. MiR-185-5p inhibitor enhanced the recruitment of microspheres into the macrophages (*,P <0.05; **, P <0.01).

Subsequent analysis was performed to clarify whether miR-185-5p mimic diminishes lipid accumulation *in vitro*. After exposure to ox-LDL for 48 h, the number of lipid droplets in macrophages was increased, as evidenced by Oil Red O staining. Co-treatment with miR-185-5p mimic and ox-LDL significantly decreased the number of lipid droplets in contrast with the findings in miR-185-5p NC group. Additionally, miR-185-5p inhibitor markedly increased the number of lipid droplets in macrophages (Figure 5A). The quantitative analysis revealed that the percentage of Oil Red O positive cells was lower in miR-185-5p mimic group than that in miR-185-5p NC and inhibitor groups. The microsphere phagocytosis assays were performed to investigate the effect of miR-185-5p on the efferocytosis capacity of macrophages. The microsphere content in the ox-LDL-stimulated macrophages was quantified. It was shown that significantly fewer fluorescent microspheres infiltrated into the macrophages in miR-185-5p mimic group. The arteriosclerotic environment led to accelerated recruitment of microspheres into the macrophages, whereas such recruitment was significantly restrained by miR-185-5p mimic. On the contrary, miR-185-5p inhibitor enhanced the recruitment of microspheres into the macrophages. The quantitative analysis confirmed that the infiltration of fluorescent microspheres was weaker in miR-185-5p mimic group compared to that in miR-185-5p inhibitor group and miR-185-5p NC group (P<0.05). These results implied that efferocytosis may be impaired by miR-185-5p mimic, potentially contributing to inhibition of lipid accumulation in ox-LDL-stimulated macrophages.

## DISCUSSION

In this study, the effects of miR-185-5p on attenuating arteriosclerosis and lipid accumulation were explored using HFD-fed apoE^-/-^ mice and ox-LDL-stimulated macrophages. Our findings indicated that miR-185-5p mimic diminished plaque formation in descending arteries and reduced the lesion areas of aortic root tissues. At the molecular level, miR-185-5p decreased the expressions of lipid metabolism-related genes via inhibiting SREBP-2 activation thus confirming the critical role of miR-185-5p for clinic use as a lipid-regulating agent in treating arteriosclerosis and related lipid metabolism disorders.

It was demonstrated through bioinformatics analysis that SREBP-2 was involved in the pathogenesis of arteriosclerosis. The biological prediction analysis showed that miR-185-5p could bind to SREBP-2, and there was a targeting relationship between miR-185-5p and SREBP-2. The GO annotations and KEGG pathway analysis were conducted on the data from GSE132651 dataset. It was revealed that the DEGs were enriched in the lipid metabolic process. This conclusion hypothesizes that the effects of miR-185-5p and its target gene SREBP2 on atherosclerosis may be achieved through the lipid metabolic signaling pathway.

To examine the regulatory effect of miR-185-5p on atherosclerosis, *in vivo* experiments were conducted on the HFD-fed apoE^-/-^ mice treated with miR-185-5p mimic and NC. MiR-185-5p is a powerful cholesterol modulator, especially in hepatic lipid metabolism, which can effectively suppress the expression of hepatic SREBP2 as well as its downstream genes LDLR and SR-BI, thereby preventing the serum LDL from entering r into hepatocytes for further hepatic lipid accumulation, and possibly leading to increased levels of serum LDL and free fatty acids [25, 26], Besides, the results in this study uncovered that the serum TC and TG levels were elevated with the decreased hepatic cholesterol level in miR-185-5p mimic group in contrast with those in miR-185-5p NC group, clearly confirming that miR-185-5p inhibits the progression of arteriosclerosis by suppressing lipid metabolism in macrophage [30–34]. The Oil Red O staining revealed that miR-185-5p mimic diminished plaque formation in descending arteries of apoE^-/-^ mice. To further evaluate the effect of miR-185-5p on atherosclerosis, Oil Red O staining was conducted to the longitudinally dissected aorta, and it was demonstrated that miR-185-5p mimic decreased the lesion area and plaque ratio in HFD-fed apoE^-/-^ mice. Moreover, arteriosclerosis is considered as a type of hyperlipidemia induced by inflammatory diseases, the lipid accumulation in macrophages activates SREBP2 and related NLRP3, a major inflammatory stimulator able to amplify the inflammatory response and result in the deterioration of arteriosclerosis [35]. The lipid metabolism and inflammation involved in arteriosclerosis in this study could be attributed to the modulation of SREBP2 by miR-185-5p.

Next, the effects of miR-185-5p on lipid metabolism at the molecular level were examined. The expression of major regulators involved in lipid metabolic processes was evaluated. The GSEA on GSE132651 dataset revealed that enrichment of lipid metabolism and regulation of epidermis development appeared to be functionally enriched in SREBP2 signaling activation (Figure 3A, 3B). Furthermore, co-expression analysis revealed that LDLR and Fas were significantly correlated with SREBP2 in atherosclerosis. Q-PCR, Western blotting analysis implied that HFD increased the mRNA and protein levels of LDLR, Fas, SREBP2, SCD-1, and HMGCR in apoE^−/−^ mice, whereas miR-185-5p mimic suppressed such expressions. These results were consistent with the bioinformatics analysis results. These regulatory proteins of cholesterol biosynthesis are responsible for the accumulation of LDL cholesterol into lesional macrophages [28]. The results of the present study uncovered that miR-185-5p inhibited the Oil Red O stained areas/lipid accumulation areas by inhibiting SREBP-2 associated proteins.

To further confirm the mechanisms of suppressive effect of miR-185-5p mimic on SREBP2 activation, macrophages were treated with ox-LDL to create an atherosclerotic environment *in vivo*. Immunofluorescence staining revealed that the administration of miR-185-5p mimic significantly down-regulated the expression of SREBP2, while the decreased expression of SREBP2 was recovered by miR-185-5p inhibitor. Western blotting analysis confirmed that miR-185-5p mimic reduced the protein expression of SREBP2 and its associated genes LDLR, and FAS, SREBP1c, SCD-1, and HMGCR *in vitro*. The results were further supported by quantitative analysis on relative protein levels, suggesting that miR-185-5p mimic specifically affects the activation of lipid metabolism-related genes in ox-LDL-stimulated macrophages. SREBP2-OE effectively reversed the protective role of miR-185-5p, namely, inhibiting its lipid metabolism-related genes via the SREBP2 activation. In summary, miR-185-5p suppressed the lipid deposition in lesional macrophages by inhibiting SREBP2 and its associated lipid modulatory proteins in the case of atherosclerosis.

In addition, Oil Red O staining revealed that miR-185-5p mimic significantly decreased the number of lipid droplets in ox-LDL-stimulated macrophages. The microsphere phagocytosis assays further confirmed that the arteriosclerotic environment led to accelerated recruitment of microspheres into the macrophages, but such recruitment was significantly restrained by miR-185-5p mimic, implying that efferocytosis may be impaired by miR-185-5p mimic, potentially contributing to the inhibition of lipid accumulation in ox-LDL-stimulated macrophages.

In conclusion, the present study provides evidence that the repression of the lipid metabolism signaling pathway induced by SREBP2 down-regulation underlies the suppressive role of miR-185-5p in atherosclerosis progression. These conclusions indicate a translational value of miR-185-5p as a novel therapeutic strategy for atherosclerosis.

## Supplementary Material

Supplementary Table 1
