# Supplementary material for: The elevation of miR-185-5p alleviates high-fat diet-induced atherosclerosis and lipid accumulation in vivo and in vitro via SREBP2 activation
Source: Aging (Albany NY). 2022 Feb 16;14(4):1729–42. doi: 10.18632/aging.203896 (PMC8908921; doi:10.18632/aging.203896)
Supplement: Supplementary Table 1 [file aging-14-203896-s001.pdf]

SUPPLEMENTARY TABLE

Supplementary Table 1. Clinicopathological characteristics of atherosclerotic patients (*,*P <0.05; **, P <0.01*).

Characteristics	Stable group	Vulnerable group
Age	64.25±3.038 N=4	69.50±2.630 N=4
Gender	Male:3/Femal:1	Male:3/Femal:1
Have hyperlipidemia	N=4	N=4
Have diabetes	N=1	N=2
Have hypertension	N=2	N=3
Maximum diameter under ultrasonic(mm)	1.098 ±0.093 N=4	3.283 ?0.2463 N=4**

Abbreviation: SREBP2, Sterol response element-binding protein 2; miRNA, micro RNAs; GO, Gene Ontology; KEGG, Kyoto Encyclopedia of Genes and Genomes; HFD, high-fat diet; ox-LDL, oxidized low-density lipoprotein.
